# Patients’ Expectations Impact Their Satisfaction following Total Hip or Knee Arthroplasty

**DOI:** 10.1371/journal.pone.0167911

**Published:** 2016-12-15

**Authors:** Audrey Neuprez, Jean-Pierre Delcour, Firouzeh Fatemi, Philippe Gillet, Jean-Michel Crielaard, Olivier Bruyère, Jean-Yves Reginster

**Affiliations:** 1 Department of Public Health, Epidemiology and Health Economics, University of Liege, Belgium; 2 Physical Medicine and Sport Traumatology Service, Motility Sciences Department, CHU, Liege, Belgium; 3 Department of Orthopaedic Surgery, CHBAH, Seraing, Belgium; 4 Department of Orthopaedic Surgery, CHC, Liege, Belgium; 5 Department of Orthopaedic Surgery, CHU, Liege, Belgium; Harvard Medical School/BIDMC, UNITED STATES

## Abstract

**Introduction:**

The objective of this study was to assess the number and magnitude of preoperative expectations and to correlate them with the degree of satisfaction expressed one year after Total Hip Arthroplasty (THA) or Total Knee Arthroplasty (TKA), in patients with severe and painful osteoarthritis (OA).

**Materials and Methods:**

Preoperative expectations (within 20 days prior to surgery) and postoperative satisfaction (one year after the intervention) were measured using the previously validated French version of the Hospital for Special Surgery Hip or Knee Replacement Expectations Survey. Postoperative satisfaction was measured using a specific scale, following the same methodology as that used for the assessment of expectations. Prediction of the satisfaction of the patients was performed using multivariate linear regression modelling.

**Results:**

A total of 138 patients (80 THA and 58 TKA) completed the two parts of the study. The expectations score (mean ± SD) (range 0–100) was 72.58 ± 12.63 before THA and 69.10 ± 13.72 before TKA (p = 0.13). The number of expectations expressed was 14.34 ± 1.32 (out of a potential maximum of 18) before THA and 14.70 ± 2.29 (out of a potential maximum of 19) before TKA. After 1 year, THA generated a significantly higher degree of satisfaction compared to TKA (69.70 ± 14.46 v 60.44 ± 17.54, p<0.001) (range 0–100). The pre-operative expectations score was the single best positive predictor of the post-surgery satisfaction assessment both for TKA and THA.

**Conclusion:**

Patients undergoing total joint arthroplasty for end-stage OA have a high level of expectations, before both THA and TKA. While both types of interventions significantly improve essential and non-essential activities, the rate of satisfaction is significantly greater post THA. Preoperative expectations are a major contributor to the final degree of satisfaction, one year after surgery. These results re-emphasize the need for an optimal preoperative interaction between health care providers and patients, to allow patients a chance to foresee a reasonable outcome after TJA.

## Introduction

The number of total joint arthroplasty (TJA) procedures of the lower limbs has dramatically increased over recent decades, and this number is expected to grow. For instance, in Sweden the estimated number of total knee arthroplasty (TKA) per 100.000 residents aged 40 years and older is 334 for the year 2020, while the projected incidence of TKA, in this community reaches 469 per 100.000 subjects in 2130 [1]. In the UK, the number of total hip arthroplasty (THA) and TKA procedures performed in 2035 is estimated to double compared to the numbers from 2010 [2]. A similar trend is observed in New Zealand, where by 2026, the number of THA and TKA procedures is expected to increase by 84% (THA) and 183% (TKA) [3]. By 2020, the projected demand for primary total hip and knee arthroplasty in the United States will exceed 1.8 million procedures, a nearly three-fold increase from the number of procedures performed in 2010 [4].

The success of the intervention is primarily based on technical parameters, which can be objectively measured. The perception of the patients’ outcomes, including the fulfilment of their preoperative expectations and the degree of satisfaction that they express after surgery are also of critical importance [5, 6]. These patients-reported outcomes are major indicators of success when assessing the outcomes of orthopedic surgery from a patient-centered perspective [7]. As anticipated, meeting patients expectations strongly correlates with the level of satisfaction that they express after surgery [5, 8]. Some studies suggest a correlation between the level of preoperative expectations and the improvement of functional outcomes after TKA and THA, but this remains controversial [9]. The percentage of patients who are satisfied after TKA knee arthroplasty is estimated to be in the range of 20% while figures between 7 and 15% have been reported for THA [10–12].

The objective of the present study is to assess the level of patients’ preoperative expectations and to correlate this parameter with the degree of satisfaction expressed after THA or TKA, in a population of patients whose surgical indication was severe and painful osteoarthritis (OA).

## Materials and Methods

Patients were recruited within the Departments of Orthopaedic Surgery in three separate hospitals located in the vicinity of the city of Liege (Belgium) and invited to take part in a prospective longitudinal study. These patients were part of the population included in our previous publication describing the validation process of the French translation of the questionnaire developed by the Hospital for Special Surgery (New York) (HSS) to assess patients’ expectations before TKA or THA for OA [13]. Details of the recruitment process were extensively described previously [13]. Briefly, OA patients scheduled for immediate TKA or THA, aged 18 years or older were contacted by phone within 20 days before surgery (first part of the study).Exclusion criteria were surgery for indications other than OA, revision surgery, lack of oral consent and language problems (comprehension or expression). After receiving a detailed explanation of the content and objectives of the study, they were given the possibility to participate, and if consent was obtained, a more exhaustive telephone interview was scheduled. At that time, information on their age, gender, height and weight (BMI), educational level and living situation was collected. The study was approved by the local medical ethical committee (University Hospital Liège (707)—Approval number: B70720084766). An oral informed consent was obtained by AN, the physician who was not directly part of the surgical management team (i.e., not a direct care provider). The same physician (AN) was also responsible for conducting all phone surveys at both times points. A specific note was included in all files confirming that this agreement was obtained. The Chairman of the Ethical Committee was informed for and approved this oral procedure, which aimed to prevent the patients from specifically coming to the hospital to sign a written informed consent, when no visit was scheduled for another purpose. To assess patients’ preoperative expectations before TKA or THA, we used two questionnaires, that had been previously translated and validated in French: the HSS Hip Replacement Expectations Survey and the HSS Knee Replacement Expectations Survey. These 18-item (hip) and 19-item (knee) surveys include 5 different categories of expectations: pain, walking, psychological state, essential activities and non-essential activities [13].

Patients were asked how much relief or improvement they expected for each item as a result of their hip/knee arthroplasty on a 5-point Likert scale.

The following ordinal response format was used:

- 4: “back to normal or complete improvement” (corresponding to very important expectation)
- 3: “a lot of improvement” (important expectation)
- 2: “a moderate amount of improvement” (somewhat important expectation)
- 1: “a little improvement” (minimally important expectation)
- 0: “I do not have this expectation, or this expectation does not apply to me”

The total score ranged from 0 to 72 for hip and from 0 to 76 for knee, and was transformed into a 100-point scale, with a higher score representing higher expectations.

One year after surgery (second part of the study), patients were again interviewed by telephone, and they were asked about the degree of satisfaction (expectations fulfilment) they had as a consequence of their THA and TKA, based on their previously expressed expectations.

Satisfaction was expressed, on a 4-point Likert scale, as:

- 3: “complete satisfaction”
- 2: “partial satisfaction”
- 1: “dissatisfaction”
- 0: “not applicable”

If the patient answer at the time of the first interview, assessing his/her expectations, was 0, for one or several items, i.e., “I do not have this expectation, or this expectation does not apply to me”, the related item(s) was/were discarded from the satisfaction questionnaire.

The total score was calculated using the same methodology, ranging from 0 to 54 for hip and from 0 to 57 for knee, and translated into a 100-point scale, with a higher score representing higher satisfaction.

The data were analyzed using STATISTICA, version 12, in a Windows environment. The level of statistical significance was set at p < 0.05 for all analyses After testing the normality and homogeneity of the data with the Shapiro–Wilk and Levene tests, respectively, participant characteristics and data one preoperative expectations and satisfaction after 1 year were assessed using descriptive statistics (frequencies and mean/standard deviation [SD] values). For the comparison between the 2 groups (THA/TKA), in the case of continuous data, Student’s t-test was performed. Categorical variables were compared with the Chi-squared test.

No formal sample size calculation was conducted. However, we based our assessment of the number of patients needed to be included in the sample that was needed for the development and validation of the French version of the HSS Expectations Surveys [13]. This number was also consistent with the number of subjects included in the validation and translation of the questionnaires in Dutch [14]. Univariate and multivariate linear regression models were created with satisfaction as the dependent variable and demographic characteristics and expectation scores as the independent variables. A significance level of 0.2 was used to include variables in the stepwise regression model and a level of 0.05 was used to determine statistical significance in the final model. R^2^ was calculated to assess the proportion of variance explained by the model.

## Results

Our initial potential cohort included 185 patients, 42 of whom we were unable to reach, and who never responded to our solicitations. Therefore, the remaining143 patients were invited to complete the first interview (i.e., assessment of their expectations) prior to surgery. All agreed to participate in this survey. Of these patients, 5 were lost to follow-up (3 deaths and 2 who did not undergo surgery because of unexpected medical circumstances). One year after the surgical procedure, 138 patients (80 THA and 58 TKA) participated in the second part (interview regarding their degree of satisfaction) of the study. Demographic characteristics, expectations and satisfaction are listed in Table 1.

**Table 1:** Number and magnitude of expectations before THA and TKA, satisfaction scores one year after THA and TKA, and patient characteristics at baseline.

	Hip	Knee	p-value
N	80	58	
Sex	50 ♀ 30 ♂	35 ♀ 23 ♂	0.80
Age (years) (mean, SD)	64.43 ± 11.28	68.10 ± 9.19	***<0*.*05***
BMI (kg/m^2^) (mean, SD)	26.69 ± 5.14	28.72 ± 4.79	***<0*.*05***
Side (Left or Right)	44 L 36 R	25 L 33 R	
Living situation			0.57
Living alone (N, %)	20 (25)	17 (29.31)	
Living with partner and/or children (N, %)	60 (75)	41 (70.69)	
Highest educational level			0.95
Primary school (N, %)	7 (8.75)	5 (8.62)	
Secondary school (N, %)	38 (47.50)	26 (44.83)	
Higher education (N, %)	35 (43.75)	27 (46.55)	
Operation performed in:			
University Medical Center (N, %)	20 (25)	20 (32.79)	
General Hospital (public) (N, %)	23 (28.75)	20 (32.79)	
General Hospital (private) (N, %)	37 (46.25)	21 (34.42)	
Expectation Score: 0–100 (mean, SD)	72.58 ± 12.63	69.10 ± 13.77	0.13
Number of expectations (mean, SD)	14.34 ± 1.92	14.70 ± 2.23	0.29
Satisfaction Score: 0–100 (mean, SD)	69.70 ± 14.46	60.44 ± 17.54	***<0*.*001***

### Number and Importance of Expectations

**Fig 1:**
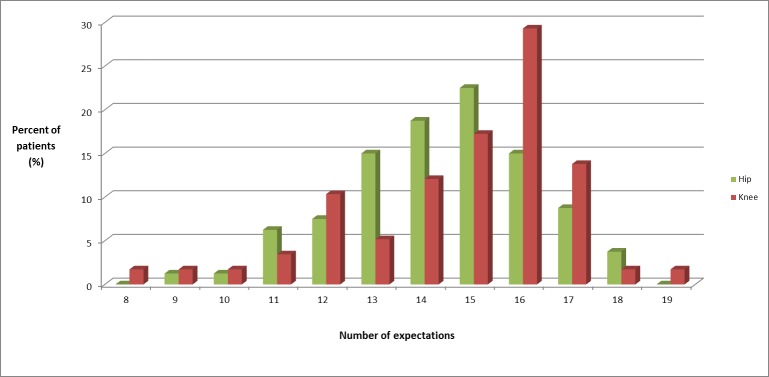
Number of expectations. Percentage of patients expressing each number of expectations from the 18-item/19-item Hospital for Special Surgery Total Hip/Knee Replacement Expectations Survey.

The expectation scores (Table 1) were similar in the 2 groups. The median expectation score was 14.5 (maximum 18) for THA and 15 (maximum 19) for TKA; 9% of the patients had expectation scores of 11 or less (Fig 1).

The majority of the expectation scores were 3 or 4, indicating they were considered as “important” or “very important” by the patients.

**Fig 2:**
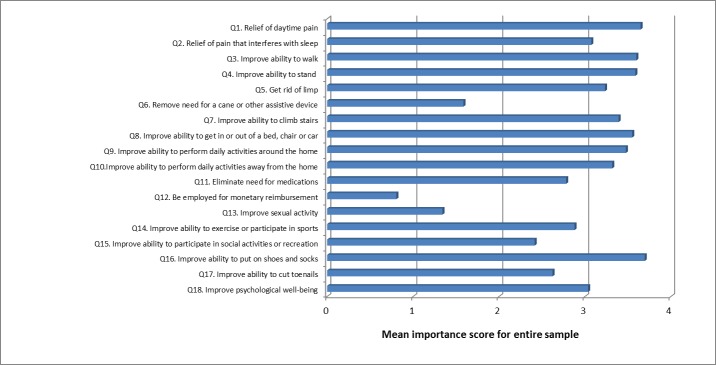
Importance of expectations (THA). Mean score for each of the 18 items of the Hospital for Special Surgery Total Hip Replacement Expectations Survey.

In patients undergoing THA, 11 of the 18 expectations were ranked with a mean score of 3 or more, i.e., perceived at least as “important” (Fig 2). The 7 remaining items, scoring with a mean value below 3 were:

- “remove need for a cane or other assistive device”,
- “eliminate need for medications”,
- “be employed for monetary reimbursement”,
- “improve sexual activity”,
- “improve ability to exercise or participate in sports”,
- “improve ability to participate in social activities or recreation”,
- “improve ability to cut toenails”.

Some items were given a score of “0” (i.e., I do not have this expectation) by a large percentage of patients because they were not applicable to the patient’s preoperative situation (57.5% did not need a walking aid, 25% did not require pain relief medications, 76% were unemployed, 62.5% did not report sexual activity, 16% did not exercise, 31% had no social or recreational activities, and 26% did not cut their toenails by themselves). Pain relief, improvement in walking, standing and ability to put on shoes and socks were the most prevalent expectations, expressed by 99% of patients. Of all the items, the expectation of an improvement in putting on shoes and socks as well as the expectation of pain relief were the most often rated as very important, i.e., by 76 and 74% of patients, respectively (Table 2).

**Table 2:** Preoperative expectations and postoperative satisfaction in THA and TKA.

Survey Items	Baseline expectations						Satisfaction		
	“Back to normal/CompleteImprovement” (%)	“A lot ofimprovement” (%)	“Moderateimprovement” (%)	“Littleimprovement” (%)	TOTAL (%)	“No expectation” (%)	Complete (%)	Partial (%)	Dissatisfaction (%)
**THA (n = 80)**									
Q1. Daytime pain	73.75	21.25	3.75	0	**98.75**	**1.25**	76.92	20	3.08
Q2. Pain during sleep	62.50	20	0	0	**82.5**	**17.5**	85.07	13.43	1.49
Q3. Walk	68.75	23.75	6.25	0	**98.75**	**1.25**	64.56	31.65	3.80
Q4. Stand	70	23.75	3.75	1.25	**98.75**	**1.25**	73.42	25.32	1.27
Q5. Rid of limp	63.75	20	3.75	1.25	**88.75**	**11.25**	61.43	34.29	4.29
Q6. Assistive device	32.50	8.75	1.25	0	**42.5**	**57.5**	60	28.57	11.43
Q7. Climb stairs	67.50	18.75	6.25	1.25	**98.75**	**6.25**	69.33	25.33	5.33
Q8. Get in or out of a bed, chair or car	68.75	25	2.50	0	**96.25**	**3.75**	74.36	23.08	2.56
Q9. Activities around the home	71.25	16.25	6.25	2.50	**96.25**	**3.75**	61.84	31.58	6.58
Q10. Activities away from the home	65	18.75	7.50	1.25	**92.5**	**7.5**	69.33	26.67	4
Q11. Medications	57.50	13.75	2.50	1.25	**75**	**25**	72.41	24.14	3.45
Q12. Monetary reimbursement	17.50	1.25	3.75	1.25	**23.75**	**76.25**	75.95	17.72	6.33
Q13. Sexual activity	30	3.75	2.50	1.25	**37.5**	**62.5**	67.74	22.58	9.68
Q14. Exercise	50	22.5	11.25	0	**83.75**	**16.25**	59.70	32.84	7.46
Q15. Social activities or recreation	41.25	20	7.50	0	**68.75**	**31.25**	69.09	23.64	7.27
Q16. Put on shoes and socks	76.25	20	2.50	0	**98.75**	**1.25**	65.82	25.32	8.86
Q17. Cut toenails	52.50	17.50	2.50	1.25	**73.75**	**26.25**	44.07	40.68	15.25
Q18. Psychological well-being	68.75	8.75	1.25	2.50	**81.25**	**18.75**	76.92	20	3.08
**TKA (n = 58)**									
Q1. Pain	70.69	25.86	3.45	0	**100**	**0**	51.72	36.21	12.07
Q2. Walk short distance	58.62	27.59	1.72	1.72	**89.66**	**10.34**	75	19.23	5.77
Q3. Walk medium distance	56.90	31.03	1.72	0	**89.66**	**10.34**	53.85	28.85	17.31
Q4. Walk long distance	53.45	36.21	51.17	1.72	**96.55**	**3.45**	41.07	25	33.93
Q5. Need for a cane, crutch or walker	27.59	3.45	0	1.72	**32.76**	**67.24**	57.89	26.32	15.79
Q6. Knee or leg straight	68.62	10.34	1.72	0	**70.69**	**29.21**	85.37	12.20	2.44
Q7. Go up stairs	68.97	18.97	5.17	1.72	**94.83**	**5.17**	56.36	38.18	5.45
Q8. Go down stairs	62.07	25.86	5.17	3.45	**96.44**	**3.45**	32.14	58.93	8.93
Q9. Kneel	34.48	29.31	25.86	3.45	**93.10**	**6.90**	5.56	35.19	59.26
Q10. Squat	37.93	32.76	20.69	1.72	**93.10**	**6.90**	22.22	48.15	29.63
Q11. Use public transportation, drive	51.72	17.24	5.17	0	**74.14**	**25.86**	65.12	30.23	4.65
Q12. Monetary reimbursement	8.62	3.45	0	0	**12.07**	**87.93**	71.43	28.57	0
Q13. Recreation	55.17	27.59	3.45	1.72	**87.93**	**12.07**	65.50	31.25	6.25
Q14. Daily activities	63.79	17.24	3.45	0	**84.48**	**15.52**	59.18	30.61	10.20
Q15. Exercise	53.45	22.41	8.62	0	**84.48**	**15.52**	48.98	26.53	24.49
Q16.Change position	68.97	17.24	3.45	0	**89.66**	**10.34**	48.08	38.46	13.46
Q17.Interact with others	62.07	13.79	6.90	0	**82.76**	**17.24**	56.25	31.25	12.50
Q18.Sexual activity	15.52	10.35	1.72	0	**27.59**	**72.41**	43.75	37.50	18.75
Q19. Psychological well-being	63.79	8.62	0	0	**72.41**	**27.59**	71.43	16.67	11.90

**Fig 3:**
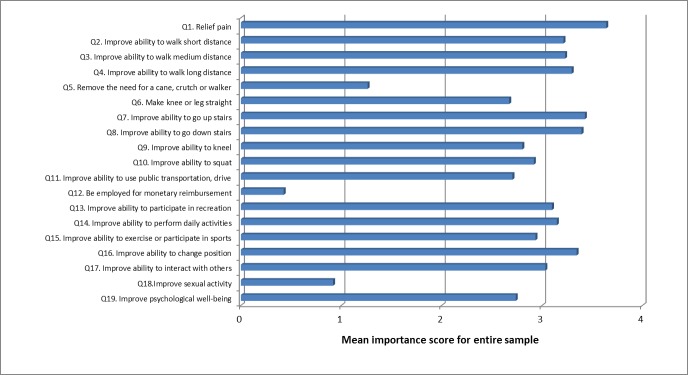
Importance of expectations (TKA). Mean score for each of the 19 items of the Hospital for Special Surgery Total Knee Replacement Expectations Survey.

In patients undergoing TKA, 10 of the 19 expectations were ranked with a mean score of 3 or more, i.e., perceived at least as “important” (Fig 3). The 9 remaining items, scoring with a mean value below 3 were:

- “Remove need for a cane, crutch or walker”,
- “Make knee or leg straight”,
- “Improve ability to kneel”,
- “Improve ability to squat”,
- “Improve ability to use public transportation, drive”,
- “Be employed for monetary reimbursement”,
- “Improve ability to exercise or participate in sports”,
- “Improve sexual activity”,
- “Improve psychological well-being”.

Some items were given a score of “0” (i.e., I do not have this expectation) by a large percentage of patients because they were not applicable to patient’s preoperative situation (67% did not need a walking aid, 29% did not need to extend their knee or leg, 26% did not need to improve their ability to use public transportation or to drive, 88% were unemployed, 16% did not exercise, 72% did not report any sexual activity, and 28% had no expectation about psychological well-being). Pain relief, improvement in walking long distance and ability to go down the stairs were the most prevalent expectations, expected by 100% and 97% of patients. Out of all expectations, pain relief was the most frequently rated as very important, by 71% of patients. Furthermore 34 and 38% of patients, respectively, expected a complete improvement in kneeling and in squatting following TKA, whereas 59 and 55% expected a partial improvement of these functions (Table 2).

### Satisfaction with the fulfilment of preoperative expectations

The mean satisfaction score reflecting the fulfilment of the preoperative expectations was significantly higher one year after THA than one year after TKA (Table 1). The degree of satisfaction expressed one year after surgery for each of the preoperative expectations is shown in Table 2. Following THA, the 5 expectations that generated the highest satisfaction rates (>74% of complete satisfaction) were night and day pain relief, a psychological benefit, a return to work and the ability to change position. Conversely, the lowest rates of complete satisfaction (<62%) were found for the expectations in improvement to cut toenails, to return to a sport activity or an activity around the home, to eliminate limping and to be able to walk without a cane. Following TKA, the 4 highest rates of complete satisfaction (>71%) were found for the expectation of an improvement in the mobility of the knee, a greater ability to walk over a short distance, a return to work and a psychological benefit from surgery, whereas the 4 lowest rates of complete satisfaction were found for the expectations of an improvement in kneeling and squatting (6% and 22%,respectively), to get down the stairs (32%), to walk a long distance (41%) and to improve sexual activity (44%).

### Relationship between preoperative expectations and satisfaction expressed one year after surgery

In the multivariate analyses, parameters recorded before surgery that were significantly associated with the patient’s overall postoperative satisfaction score were the overall expectation score (B = 0.55, 95% CI 0.33 to 0.77, p <0.001) and the patient’s educational level (B = 3.48, 95% CI 0.74 to 6.22, p = 0.013) in the THA group and the overall expectation score (B = 0.72, 95% CI 0.49 to 0.95, p <0.001) in the TKA group. Higher expectation scores (and higher education level in the THA group) predicted higher satisfaction. These parameters explained 27% (THA group) and 41% (TKA group) of the variance of the patient’s satisfaction score (Table 3).

**Table 3:** Prediction of a higher satisfaction score for THA and TKA on multivariable analysis (final model).

Positive predictors in the THA model (r^2^ = 0.27)	B (95% confidence interval)	p-value
Expectation Score	0.54 (0.32 to 0.76)	<0.001
Highest educational level	5.32 (0.99 to 9.64)	0.02
Positive predictors in the TKA model (r^2^ = 0.41)	B (95% confidence interval)	p-value
Expectation Score	0.72 (0.49 to 0.95)	<0.001

## Discussion

The HSS Hip and Knee expectations surveys were designed to summarize the most meaningful patient expectations before Hip and Knee TJA [5, 6, 13]. They provide information on the expectations of the individual patient based on his/her particular situation, lifestyle and needs. One of the objectives is to allow a personal interaction between the patient and the surgeon to discuss every expectation in detail. Indeed, it would be genuinely expected that patients who are more severely affected in different dimensions of health-related quality of life, with a major impairment in their activities of daily living have greater expectations. However the situation is less straightforward since some studies reported higher expectations for patients with a better preoperative function [15, 16], whereas others showed an inverse correlation between preoperative functional status and patients’ expectations [17, 18].

Our study showed that OA patients requiring TJA have a high level of expectations before surgery. These expectations are similar in number and magnitude for both TKA and THA patients. However, the level of satisfaction observed one year post-surgery is higher following THA than after TKA. The degree of preoperative expectations is positively correlated with the satisfaction with TJA, for both hip and knee. Preoperatively, the majority of our patients had 15 or more expectations, with 11 items (out of 18) and 10 items (out of 19) classified as “important” in the THA and in the TKA groups, respectively. This means that the vast majority of our patients did indeed express a high degree of expectations before undergoing TJA in terms of pain, walking, psychological state, essential activities, and nonessential activities.

This preoperative information is of great importance for the surgeon because patients with unrealistically high expectations are potentially non-adherent to postoperative recommendations, and this inadequate degree of expectations, if not identified and appropriately managed by the surgeon, is a strong predictor of poor surgical outcomes [6, 18]. We know that most patients have higher expectations compared to their surgeons. Surgeons and patients often have discrepant degrees of expectations for TJA; some activities, such as sports practice, are a classic example of unrealistic expectations from the patients. More disabled patients usually expect better outcomes than their surgeons do, and this discrepancy must be resolved prior to the intervention to avoid poor levels of postoperative satisfaction on the part of the patients [19, 20].

During the last decade patient entered outcomes took a prominent place in the assessment of medical and surgical procedures [21]. Several studies in TJA have demonstrated the importance of the fulfilment of preoperative expectations in the degree of postoperative satisfaction expressed by the patients. The HSS Hip or Knee Replacement Expectations Survey used in the present study has been repeatedly reported to be an accurate and appropriate tool to assess patients’ expectations and to predict their postoperative degree of satisfaction [22–24]. Postoperative satisfaction is critically important because it is positively related to an increase in the patient’s compliance to the surgeon’s and staff’s recommendations [5].

An important observation in our trial is that patients clearly identify items for which they have high preoperative expectations, while some other items do not represent an important potential improvement. For both TKA and THA, improvement in pain and walking ability and generating an impact on activities of daily living appear to be the most important expectations. Conversely, improvements in sexual activity, the opportunity to obtain employment and, surprisingly, to be able to walk without any assistance (i.e., cane or other device) did not appear to be major preoperative concerns for our patients. In this study, we derived from our observations, one year after surgery a satisfaction score, that was specifically related to the expectations expressed by the patients prior to surgery. This approach is original and allows for a more accurate assessment of the fulfilment of patients’ preoperative expectations. At baseline, expectations before TKA or THA did not differ significantly. However, we showed that the degree of satisfaction (partial and complete expectation fulfilment) reached after THA was greater than after TKA (94% vs 85%), whereas dissatisfaction was more often expressed after TKA than THA (15% vs 6%). This observation is consistent with the results of most previous studies [25, 26] but not all of them [27]. The main determinant of satisfaction was the level of preoperative expectations. We did not find any association between the overall satisfaction score and the patients’ socio-demographic characteristics, except for the level of education in the THA group. This observation is supported by two previous studies that also suggest that optimistic expectations could be a predictor for a higher ultimate satisfaction [8, 15].

Furthermore, an improvement in the ability to cut toe nails after THA and to walk a long distance or to squat or kneel after TKA, while being expected by a substantial proportion of patients, did not generate a high degree of satisfaction in our population, a feature that was also observed in a recently published study assessing expectations and satisfaction in patients before and after TJA. In this study, preoperative and postoperative scores were subtracted to calculate whether expectations were unfulfilled, fulfilled or exceeded, whereas we used a specific questionnaire to assess postoperative satisfaction [23].

Our study was conducted in a homogeneous cohort of patients in need of TJA as a consequence of severe OA. Unfortunately, we do not have extensive information regarding the functional capacity of the patients at the time of the intervention; notwithstanding, TJA is usually an end-stage treatment in OA, offered to patients with a painful disease negatively impacting the ability to cope with activities of daily living [28]. A better knowledge of the preoperative clinical status, extensive information on the number and severity of co-morbidities and the type of prosthesis could have allowed for a more accurate analysis of our results, since it is well described that these parameters have a significant influence on both expectations and satisfaction, as well as on the overall outcomes of the surgical procedure [5, 18, 21]. The absence of a significant association with the patient baseline score and co-morbidities would have been a robust confirmation of the results of the study. An other weakness of our study is that we do not have any purposed information on the postoperative course of the patients. Postoperative complications could have had an impact on the overall outcomes in some patients in our trial. However, in the previous trial, it was suggested that major postoperative complications do decrease the satisfaction of patients after knee arthroplasty, whereas they do not appear to have a significant impact on the rate of satisfaction at twelve months post-surgery after THA [12]. Furthermore, the surgeon was not the same for all patients, and we have no information on the content of the preoperative discussions. This would have been an asset, since information given by the medical staff has been shown to influence patients’ expectations and subsequent satisfaction [6, 22]. The choice of a telephone interview conducted by a health care professional outside of the surgical team, instead of a face-to-face meeting, can be challenging. This method was selected to mitigate the potential discomfort of participating in the study and to allow the patients to not feel influenced in their answers. We did not find any previous studies suggesting that the assessment of expectations and satisfaction may be influenced by the format of the interview.

In conclusion, we showed that patients undergoing TJA for end-stage OA have a high level of expectations before both THA and TKA. While both types of interventions significantly improve essential and non-essential activities, the rate of satisfaction is significantly greater after THA. Preoperative expectations are a major contributor to the final degree of satisfaction one year after surgery. These results re-emphasize the need for an optimal preoperative interaction between the health care providers and the patients to allow the patients a chance to foresee a reasonable outcome of the TKA. Patient and physician education and interaction will improve postoperative satisfaction, which, in turn, was shown to increase patient’s compliance with recommendations from the staff.

## Supporting Information

S1 File Data set. Data used for statistical analysis.
(XLS)
